# Combining data augmentation and domain information with TENER model for Clinical Event Detection

**DOI:** 10.1186/s12911-021-01618-3

**Published:** 2021-11-16

**Authors:** Zhichang Zhang, Dan Liu, Minyu Zhang, Xiaohui Qin

**Affiliations:** College of Computer Science and Engineering, Northwest Normal University, 967 Anning East Road, 730070 Lanzhou, China

**Keywords:** Data augmentation, Pre-trained language model, Transformer, Clinical Event Detection, Electronic medical record

## Abstract

**Background:**

In recent years, with the development of artificial intelligence, the use of deep learning technology for clinical information extraction has become a new trend. Clinical Event Detection (CED) as its subtask has attracted the attention from academia and industry. However, directly applying the advancements in deep learning to CED task often yields unsatisfactory results. The main reasons are due to the following two points: (1) A great number of obscure professional terms in the electronic medical record leads to poor recognition performance of model. (2) The scarcity of datasets required for the task leads to poor model robustness. Therefore, it is urgent to solve these two problems to improve model performance.

**Methods:**

This paper proposes a combining data augmentation and domain information with TENER Model for Clinical Event Detection.

**Results:**

We use two evaluation metrics to compare the overall performance of the proposed model with the existing model on the 2012 i2b2 challenge dataset. Experimental results demonstrate that our proposed model achieves the best F1-score of 80.26%, type accuracy of 93% and Span F1-score of 90.33%, and outperforms the state-of-the-art approaches.

**Conclusions:**

This paper proposes a multi-granularity information fusion encoder-decoder framework, which applies the TENER model to the CED task for the first time. It uses the pre-trained language model (BioBERT) to generate word-level features, solving the problem of a great number of obscure professional terms in the electronic medical record lead to poor recognition performance of model. In addition, this paper proposes a new data augmentation method for sequence labeling tasks, solving the problem of the scarcity of datasets required for the task leads to poor model robustness.

## Background

In recent years, with the development of deep learning, and the increasing demand for medical information from medical information technology applications such as medical Q & A and drug-assisted research, the use of natural language processing (NLP) technology to effectively extract medical information from electronic medical records has become vital. On account of the importance of this subject, the 2012 Informatics for Integrating Biology and the Bedside (i2b2) proposed a shared [1], which is the identification and linking of mentions of temporal expressions (TEs) (eg, dates, times, durations, and frequencies) and clinically relevant events (eg, patient’s problems, tests, treatments) in narratives. As its subtask, Clinical Event Detection (CED) has been widely studied because of its potential help in constructing clinical event lines, assisted diagnosis and other tasks.

The task of Clinical Event Detection is to identify the boundary of the event in the electronic medical record and determine its type. The event detection to identify the boundary and determine type is usually considered as a sequence labeling task. The task of Clinical Event Detection (CED) and named entity recognition (NER) belong to the sequence labeling task. Therefore, it is feasible to directly apply the advancement of NER technology to Clinical Event Detection tasks. Past research mainly includes methods based on traditional machine learning and deep learning. After 2012 i2b2 challenge task is proposed, The team involved in the task has adopted many different methods: rule-based, support vector machine (SVM) [2], conditional random field (CRF) [3], Markov Logic and some combination of these methods, the best performance is the CRF-based model proposed by Beihang University et al [1]. Roberts et al. [4] used a combination of supervised, unsupervised and rule-based method, and the task ranked third. First, it uses the CRF classifier to identify event boundaries, then use an independent SVM classifier for type detection. Kovačević et al. [5] combined rules and machine learning and achieved F1 measure of 79.85%. it proposed the event CRF models were trained on relevant (type-specific) subsets of the training data and they all shared some feature groups. Cyril et al. [6] built Random Forest models to identify event modality and polarity. The emergence of deep learning greatly reduces the difficulty of obtaining text features. Zhu et al. [7] proposed a bidirectional LSTM-CRF model is trained for clinical concept extraction using the contextual word embedding model, it achieved the best performance among reported baseline models on the i2b2 2010 challenge dataset. Recently, research on the 2012 i2b2 dataset has decreased, but the NER task has been widely studied. The LSTM and CRF models greatly improve the performance of the NER task [8]. Chen et al. [9] proposed a simple but effective CNN-based network for NER, gated relation network (GRN), which is more capable than common CNNs in capturing long-term context. graph neural networks (GNNs) are also widely used in NER tasks [10].

Bidirectional long short-term memory network (BiLSTMs) is widely employed in sequence labeling tasks owing to its high power to learn the contextual representation of words [11]. But the defect of BiLSTM is that needs to be processed sequentially over time, it cannot be calculated in parallel. As for Transformer model, it not only advantage in modeling the long-range context, but fully make use of the concurrence power of GPUs. However, the position embedding of Transformer has no direction-aware, when it is projected into the query and key space of self-attention, the property of distance-awareness also disappears. Therefor, Its performance in the sequence labeling task is not like in other fields so good. Yan et al. [12] introduced the adapting Transformer encoder (TENER) model it solved the position embedding problem. However, experiments have proved that the effect is unsatisfactory only using TENER model for CED task, Analysis of the reasons is mainly due to the lack and particularity of clinical data.

**Fig. 1:**
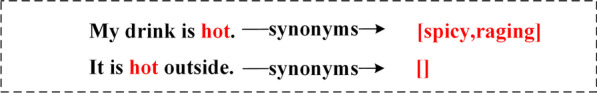
An example of the characteristics of related words in data augmentation

Data augmentation is the most widely used way to solve the problem of lack of data. Previous automatic data augmentation models are often used in speech [13] and image [14] and can help train more robust models in smaller datasets. However, the data augmentation technology in NLP has not been extensively studied. Wei et al. [15] proposed easy data augmentation (EDA) techniques for boosting performance on text classification tasks is synonym replacement, random insertion, random swap and random deletion. Zhu et al. [16] used model to generate data is a data augmentation mothed for machine reading comprehension. The data augmentation of the sequence labeling task mainly solves the problem of the imbalance of the data type samples of the NER task [17]. There are other methods, such as back translation [18], data noise as smoothing [19] and predictive language models [20]. The past text data augmentation methods are random and may change the structure of sentences. For sequence labeling tasks, if the sentence structure is changed, the previous methods will be infeasible. This paper proposes a novel data augmentation method for sequence labeling tasks, use the CheckList [21] to find replacement words to replace some words in the sentence, thereby constructing the same format sentence as the dataset. In particular, the replacement words contain sentence information, namely, corresponding the synonyms or other related words of a word are not simply searched in the dictionary, but the synonyms or other related words of the corresponding sentence. As shown in Fig. 1, using CheckList to find the synonyms word to replaceable “hot” in the sentence “My drink is hot” are “spicy” and “raging”. But using CheckList to find in the sentence “It is hot outside”, can not find the synonym word to replaceable “hot”.

Because of the particularity of clinical data, direct application of NLP advancements to clinical text mining often yields unsatisfactory results. Lee et al. [22] investigate how the recently introduced pre-trained language model BERT can be adapted for biomedical corpora, and proposed a domain-specific language representation model pre-trained on large-scale biomedical corpora (BioBERT) to solve this problem. Therefore, this article adopts the BioBERT for word-level embedding to solve the problem of poor model performance caused by the particularity of clinical data. In addition, in the past few years, in order to alleviate the data sparsity and OOV problem in word representation, Lample et al. [23], Ma et al. [24] and Liu et al. [25] have added character-level coding to the model of the English NER task and proved its effectiveness. For the Chinese NER task, in order to avoid segmentation errors, Zhang et al. [26] proposed a lattice structured LSTM model, which encodes a sequence of input characters and all potential words that match a lexicon. However, since the lattice structure is complex and dynamic, most existing lattice-based models are hard to fully utilize the parallel computation of GPUs, Li et al. [27] converted the lattice structure into a flat structure consisting of spans. The character-level features of words may show word features, for example, the beginning of “un” generally indicates negative characteristics. Therefore, adding character-level embedding will also have an impact on improving the performance of the model. Convolutional Neural Networks (CNNs) widely applied for character embedding [28], but its specified kernel size, it cannot recognize n-gram and discontinuous characters, so adopting TENER to model the character-level features in this paper [12].

In summary, the contributions of this article are as follows: (1) For the first time, we applied TENER to the CED task for multi-granularity information feature extraction. (2) We use the pre-trained language model BioBERT for word-level embedding, which effectively solves the problem of the particularity of clinical data. (3) We propose a new method for sequence labeling tasks, which effectively solves the problem of lack of datasets in the clinical field. Experimental results show that our proposed model is significantly and consistently superior to the previous state-of-the-art technology.

## Methods

**Fig. 2:**
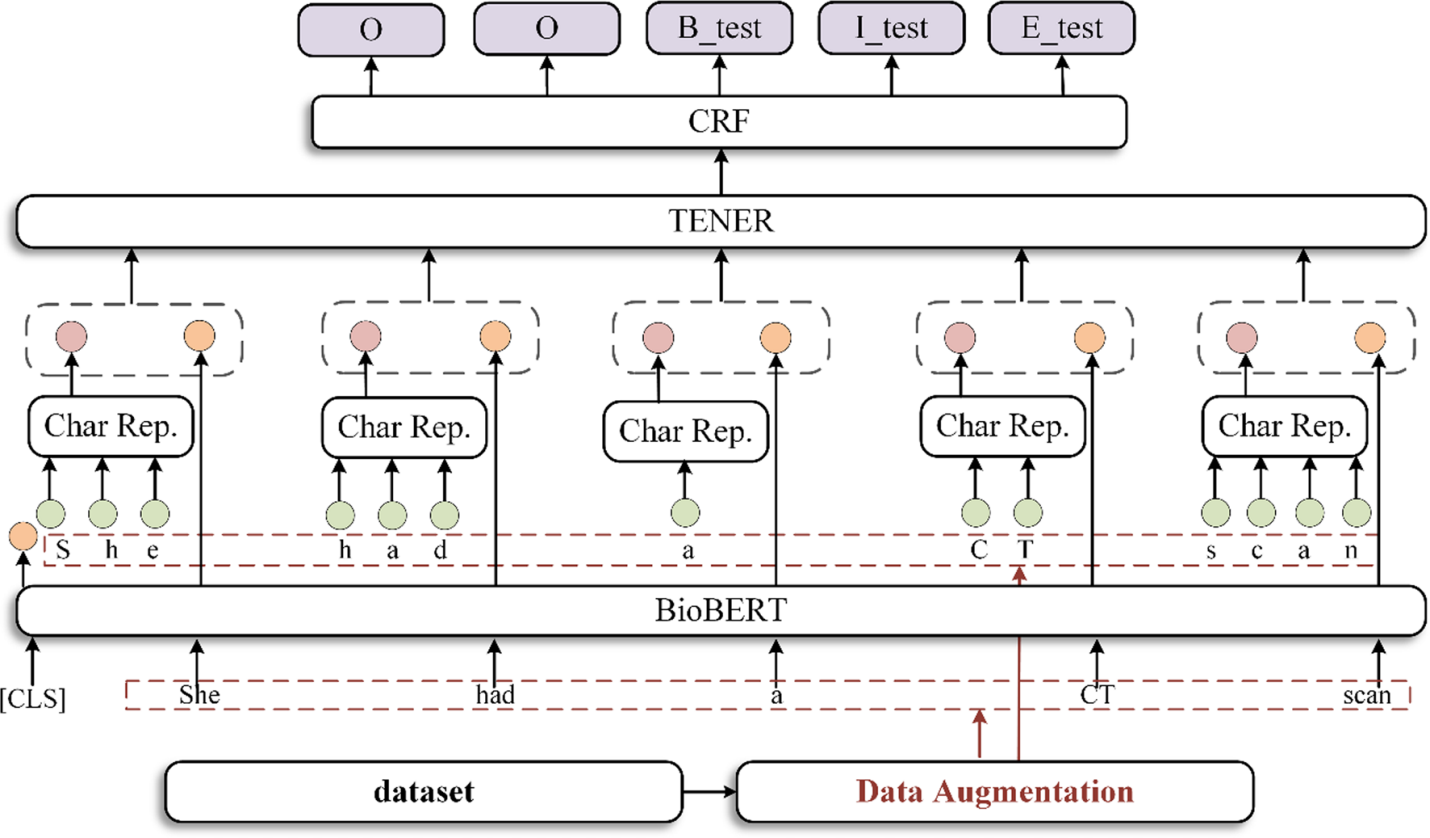
The framework of our model

This paper proposes a multi-granularity information fusion encoder-decoder framework. The model in this article mainly includes four parts: data augmentation, embedding layer, encoding layer, and decoding layer. Figure 2 illustrates an overview of our model, where “Char Rep”. is character-level encoder. First, the word-lever embedding generated by the pre-trained language model in the medical field (BioBERT) and the character-lever embedding generated by the Transformer are merged and used as the input of the encoder. Then, use the adaptive Transformer encoder (TENER) to further integrate the information. Finally, use CRF to decode and assign category labels to each word. In addition, this paper proposes a new data augmentation method, which adopts CheckList to generate a wide variety of data, improving the generalization ability of the model. We will describe each part in detail in the following sections.

### Data augmentation

**Fig. 3:**
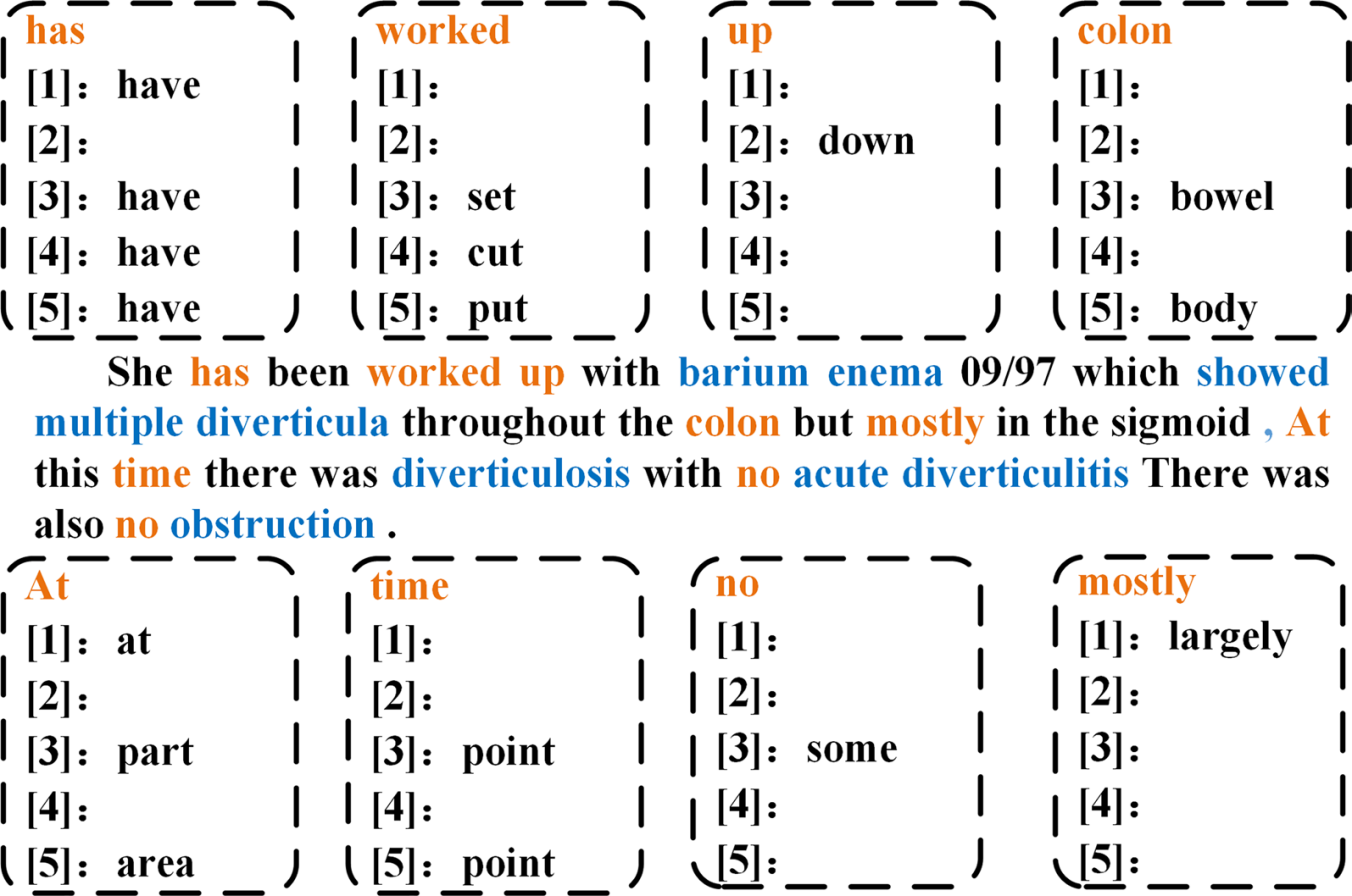
Examples of replacement words, where the words marked in blue are events, and the words marked in yellow are the replaceable words. [1] means synonyms, [2] means antonyms, [3] means hypernyms, [4] means hyponyms, [5] means related-word

The size and quality of dataset have a great impact on model performance, but it is usually cumbersome to collect. This paper proposes a new data augmentation method for sequence labeling tasks, which needs to use CheckList [21]. CheckList is a new evaluation methodology for comprehensive behavioral testing of NLP models. it guides users in what to test, by providing a list of linguistic capabilities, which are applicable to most tasks. It includes a matrix of general linguistic capabilities and test types that facilitate comprehensive test ideation, as well as a software tool to generate a large and diverse number of test cases quickly. This article uses CheckList to generate sentences with the same format as the original data, that is, only replace the non-event words in the original data, and replace them with synonyms, antonyms, hypernyms, hyponyms and related-words (hyponyms of hypernyms). For a sentence in the dataset, this article will first exclude the word representing the event in the sentence, and then use CheckList to find and replace. It mainly generates data by replacing one replaceable word and replacing all replaceable words, we think that antonyms may have a negative effect on the training of the model, so we also used the method of removing antonyms to generate new sentences. As shown in the Fig. 3, where the words marked in blue are events, and the words marked in yellow are the replaceable words. $\left[ 1\right]$ means synonyms, $\left[ 2\right]$ means antonyms, $\left[ 3\right]$ means hypernyms, $\left[ 4\right]$ means hyponyms, $\left[ 5\right]$ means related-word. The sentences in the picture are from the 2012 i2b2 challenge dataset, the hypernyms of the replaceable word “worked” corresponding to this sentence is “set”, the hyponym is “cut”, and the related-word is “put”, it does not have synonyms and antonyms. It should be pointed out that the CheckList [21] may find multiple related words, that is, there may be multiple corresponding synonyms, and there may be multiple other related words. Since constructing too many sentences may not be conducive to the training of the model, this article only uses the first relevant word to construct enough new sentences.

After finding the related words, this article replaces the replaceable words in the original sentence according to the priority order of related words (synonyms $\rightarrow$ antonyms $\rightarrow$ hypernyms $\rightarrow$ hyponyms $\rightarrow$ related-word) to generate new sentences. Since the original sentence is too long, we intercept a part of the sentence to show the constructed new sentence. Based on the original sentence “She has been worked up with barium enema 09/97.”, we constructed new sentences as shown in the Table 1, where “All” means replacing all replaceable words, “All-antonyms” means deleting antonyms and then replacing all replaceable words, “One-antonyms” means deleting antonyms and then replacing one replaceable word.

**Table 1:** Examples of data augmentation, where the bold part indicates the changed word in the sentence

Mothed	Data augmentation
All	She **have** been **set down** with barium enema 09/97.
All-antonyms	She **have** been **set** up with barium enema 09/97.
One-antonyms	She **have** been work up with barium enema 09/97.
One-antonyms	She has been **set** up with barium enema 09/97.

### Embedding layer

#### Character-level embedding

**Fig. 4:**
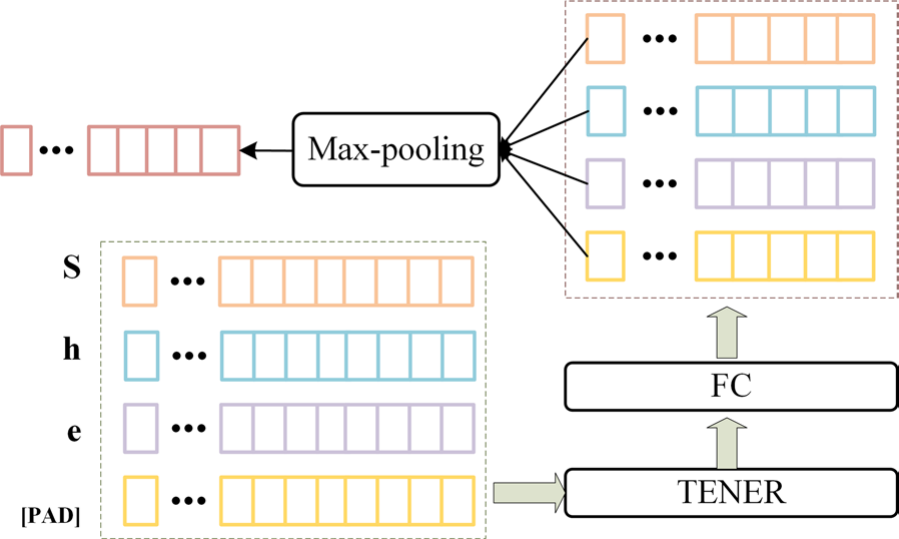
Details of Char Rep

The character-level features encoder is shown in Fig. 4, it uses TENER and fully connected network(FC) model on the character sequence $w^c=c_1,c_2 \ldots c_{wl}$ (*wl* represents the number of characters in the longest word, namely, the maximum length of the word). Each character $c_j$ is represented using:

1 $$\begin{aligned} h_j^c=e^c (c_j) \end{aligned}$$

where $e^c$ denotes a character embedding lookup table. The TENER takes in a Character-level representation matrix $H^c=\left[ h_1^c;h_2^c;\ldots ;h_{wl}^c\right] ^T$, $H^c\in R^{(wl \times cd)}$, where *cd* is the character-level embedding dimension. Then, calculate the output of the TENER layer using the equations below:

2 $$\begin{aligned} Q^h,K^h,V^h &= H^c W_q^h,H^c,H^c W_v^h \end{aligned}$$

3 $$\begin{aligned} R_{t-j} &= {\left[ \ldots \sin \left( \frac{t-j}{10000^{2i/d_k}}\right) \cos \left( \frac{t-j}{10000^{2i/d_k}}\right) \ldots \right] }^T \end{aligned}$$

4 $$\begin{aligned} {A^h}_{t,j}^{rel}&= Q_t^h{K_j^h}^T+Q_t^hR_{t-j}^T+u{K_j^h}^T+vR_{t-j}^T \end{aligned}$$

5 $$\begin{aligned} Attn(Q^h,K^h,V^h)&= soft\max ({A^h}^{rel})V^h \end{aligned}$$

6 $$\begin{aligned} {head}^h&= Attn(Q^h,K^h,V^h) \end{aligned}$$

7 $$\begin{aligned} H^{\prime} &= MultiHead(H^c)=[head^{(1)};\ldots ;head^{(n)}] \end{aligned}$$

where h represents the head index, $W_q^h$,$W_v^h\in R^{cd \times cd_k }$ are learnable parameters, $K^h$ is split *H* into $cd/{cd}_k$ partitions in the second dimension, usually $cd_k \times n=cd$, *n* is the number of heads in Eq. 7, $R_{t-j}$is the relative positional embedding, and $R_{t-j}\in R^{{cd}_k}$; *t* and *j* are index of the target token and context token, *i* is in the range $[0,d_k/2]$. in Eq. 4, ${A^h}_{t,j}^{rel}$ is attention score between two tokens with relative positional embedding, $Q_t^h {K_j^h}^T$ is the attention score between two tokens, $Q_t^hR_{t-j}^T$ is the *t* token’s bias on certain relative distance, $u\in R^{{cd} _k}$, $v\in R^{{cd}_k}$ are learnable parameters, $u{K_j^h}^T$ is the bias on the *j* token, $vR_{t-j}^T$ is the bias term for certain distance and direction. The softmax in Eq. 5 is along the last dimension. Eq. 8 means concatenation in the last dimension. Calculate the output of the FC layer using the equations below:

8 $$\begin{aligned} FFN(H^{\prime})=\max ( 0,H^{\prime}W_1+b_1)W_2+b_2 \end{aligned}$$

where $W_1\in R^{cd \times cd_{ff}}, W_2 \in R^{cd_{ff} \times cd}, b1 \in R^{cd_{ff}}, b2 \in R^{cd}, cd_{ff}$ is a hyper-parameter, $FFN(H^{\prime}) \in R^{wl \times cd}$ . In order to make the model adjustable for the character-level representation dimension of words, this paper adds a fully connected layer, and finally the character-level representation of the word $h^c$ is obtained by maximum pooling according to the penultimate dimension, $h^c \in R^{cd}$.

#### Word-level embedding

In order to solve the problem of the particularity of clinical data, this article uses BioBERT (a domain-specific language representation model pre-trained on large-scale biomedical corpora) [25]. BioBERT initializes weights from BERT, which is pre-trained on the English Wikipedia and Books Corpus general domain corpus. Then, BioBERT is pre-trained on PubMed abstract and PMC full-text article biomedical corpus. Specifically, it uses pre-trained BioBERT on PubMed for 1M steps model, this version as BioBERT v1.1 (+PubMed). Fine-tuned based on this model, the BioBERT model takes in word sequence $s=w_1,w_2\ldots w_l$ (*l* represents the maximum sentence length), Calculate the output of the BioBERT layer using the equations below:

9 $$\begin{aligned} H^w=BioBERT(s) \end{aligned}$$

where $H^w \in R^{l \times wd}$, *wd* is the word-level embedding dimension, the model limits it to a multiple of 768. This article is the last BioBERT layer, so the size is 768.

### Encoding layer

The character-level features of a sentence is expressed as $H^c=[h_1^c,h_2^c,\ldots ,h_l^c ]^T, H^c\in R^{l \times cd},$Join it and The word-level features of a sentence $H^w$ along the last dimension using the equations below, where $H^w \in R^{l \times wd}$:

10 $$\begin{aligned} H=[H^c;H^w] \end{aligned}$$

The TENER takes in a Character-level and word-level representation matrix $H, H \in R^{l \times (cd+wd)}$,the subsequent calculation is the same as the character-level TENER. We simply express it as the following formula:

11 $$\begin{aligned} H^{\prime}=TENER(H) \end{aligned}$$

Where $H^{\prime} \in R^{l \times (cd+wd)}$, In order to adapt the output dimension of TENER to the input of CRF, we added a fully connected layer. The specific calculation formula is as follows:

12 $$\begin{aligned} FC(H^{\prime})=H^{\prime}W_3+b_3 \end{aligned}$$

where ${W_1\in R}^{(cd+wd) \times d_f}, b_3 \in R^{d_f}, d_f$ is a hyper-parameter. the output dimension of the fully connected layer (FC) is $l \times d_f$, The value of $d_f$ is the number of label types.

### Decoding layer

The output of TENER layer and FC layer is expressed as $H=[h_1,h_2,\ldots ,h_l ]^T, H \in R^(l \times d_f )$, it is input to the CRF layer to predict the corresponding tag sequence. The probability of a label sequence $Y=y_1,y_2 \ldots y_l$ is

13 $$\begin{aligned} P(y|H)=\frac{\exp (\sum _i(W_{CRF}^{y_i }h_i+b_{CRF}^{(y_{i-1},y_i)}))}{\sum _{y^{\prime}}\exp (\sum _i(W_{CRF}^{y_i }h_i+b_{CRF}^{(y_{i-1},y_i)}))} \end{aligned}$$

Where $y^{\prime}$ represents an arbitary label sequence, $W_{CRF}^{y_i }$ is a model parameter specific to $y_i$, and $b_{CRF}^{(y_{i-1},y_i)}$ is a bias specific to $y_{i-1}$and $y_i$. Finally, the Viterbi Algorithm is used to find the path achieves the maximum probability.

## Results

### Dataset

**Fig. 5:**
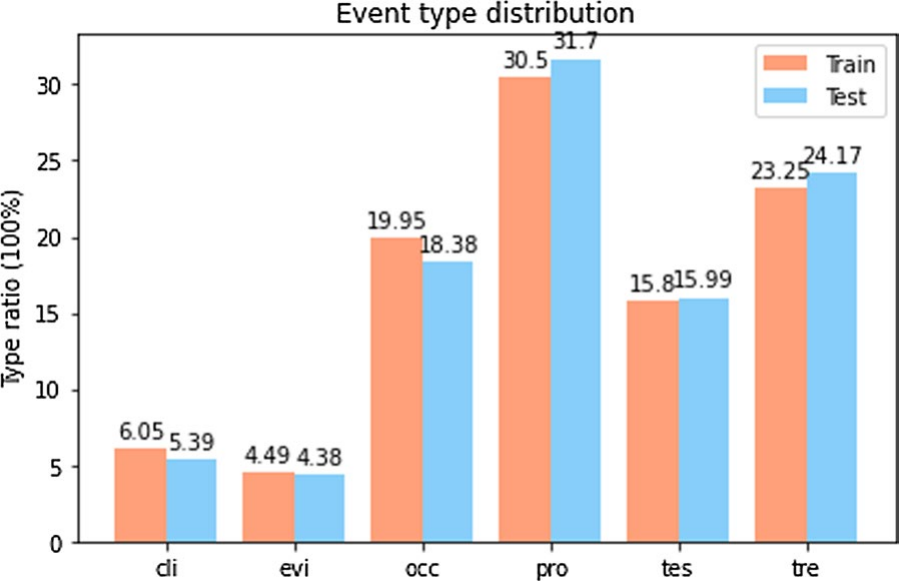
Distribution of clinical event type

To evaluate our proposed model, we experiment on 2012 i2b2 challenge dataset, the training corpus consists of 190 electronic medical records, which contains 2250 sentences (The number after adjusting the sentence length), and the test corpus of 120 electronic medical records, which contains 1741 sentences (The number after adjusting the sentence length), event types include clinical department, evidential, occurrence, problem, test, treatment. The proportion of each type is shown in the Fig. 5. The 2012 i2b2 challenge dataset does not have development set, this article divides the test set into a test set and a development set at a ratio close to 1:1. Among them, there are 821 sentences in the development set, 920 sentences in the test set.

### Evaluation metrics

This paper Clinical Event Detection is a sequence labeling task, standard precision (P), recall (R) and F1-score (F) are used as evaluation metrics, The calculation method is:

14 $$\begin{aligned} P&= \frac{TP}{TP+FP} \end{aligned}$$

15 $$\begin{aligned} P&= \frac{TP}{TP+FN} \end{aligned}$$

16 $$\begin{aligned} F &= \frac{2 \times P \times R}{P+R} \end{aligned}$$

where *TP* represents the number of events predicted as events, *FP* represents the number of non-events predicted as events, and FN represents the number of events predicted as non-events, this is strict matching, that is, span predictions must be completely consistent. In order to prove the effectiveness of the model, this article also uses the same evaluation metrics as 2012 i2b2 challenge (Span F1-score and Type accuracy) [1]. We used the Span F1-score, the harmonic mean of precision and recall of the predict output span against the gold standard span to evaluate event span detection performance. The calculation of Span F1-score is the same as the calculation of the previous F1-score evaluation metrics. It is worth noting that the Span F-score is lenient matching (predict event span overlap with the gold standard span). For event types, we calculated classification accuracy, that is, the percentage of correctly identified event types for the events whose spans are detected correctly, the specific calculation process is as follows:

17 $$\begin{aligned} P=\frac{|pred.type\bigcap glod.type|}{|pred.span\bigcap glod.span|} \end{aligned}$$

where “pred.type” means predict type output , “gold.type” means gold standard type,“ pred.span” means predict span output , “gold.span” means gold standard span.

### Experimental setup

In the process of data preprocessing, in order to solve the large gap in sentence length, we split the sentence according to several punctuation marks. such as “,”, “;” etc, these punctuation marks can be used to break the sentence, the sentence is still complete. Then join several short sentences that are adjacent but whose total length does not exceed the maximum length. Because some special characters have their own characteristics, this article will deal with them to increase accuracy, such as “’s”, splice it directly to the previous word, and we replace all digits with “0”. In addition, we use the data augmentation method of this article to expand the training set. After the training set is expanded with different degrees mothed of data, the number of sentences is distributed as shown in the Table 2. Data augmentation is performed in a way that only replaces one replaceable word, a large number of sentences will be generated, therefore, we will also randomly sample different proportions of sentences for training (20%, 40%, 60%, 80%, 100%), the number of these sentences is displayed in the Table 5 of the result analysis section.

In the experiment based on the CED task, we use the BIOES tag schema. For character-level embedding, we set randomly initialized character embedding size cd to 30, the number of Transformer layers is 1, the number of heads *n* is 3, and the dimension of the middle FC layer $cd_{ff}$ is 60. For word-level embedding we only take the last layer, the dimension is 768. For encoding layer, the number of Transformer layers is 2, the number of heads *n* is 8. the batch size for training is 16, epochs is 100, we use SGD and 0.9 momentum to optimize the model. During the optimization, we use the triangle learning rate where the learning rate rises to the pre-set learning rate (0.0008) at the first 1% steps and to 0 in the left 99% steps [29].

**Table 2:** Sentence numbers after data augmentation

Augmentation method	Number
No data augmentation	2250
Replace all replaceable words	4393
Replace all replaceable words without antonyms	4380
Replace one replaceable word without antonyms	15763

### Experimental results and analysis

#### Evaluation on CED

We compare proposed model with the latest model on the 2012 i2b2 challenge dataset. In addition, we will also apply the latest model for NER to the data set of this article for comparative experiments. The 2012 i2b2 challenge test results are shown in Tables 3 and 4.

**Table 3:** Results of Precision, Recall and F1-score metrics, where the bold part indicates the best result

Model	Precision	Recall	F1-score
Rule-based and machine learning [5]	**81.47**	78.05	79.85
BiLSTM_CRF	74.84	52.72	61.86
ELMo_TENER [12]	74.54	78.05	76.26
Ours	81.01	**79.53**	**80.26**

**Table 4:** Results of Span F1-score and Type accuracy metrics, where the bold part indicates the best result

Model	Span F1-score	Type accuracy
Beihang University et al. (CRF) [1]	**91.66**	86.00
Vanderbilt University (CRF _SVM) [1]	90.00	84.00
The University of Texas (CRF_SVM) [1]	89.00	80.00
supervised,unsupervised and rule-based [4]	89.33	80.45
TENER (the Glove 100d) [12]	74.24	75.05
Ours	90.33	**93.00**

The overall results of the model using P, R and F evaluation metrics are shown in Table 3. In the first block, the model combines rule-based and machine learning approaches that rely on morphological, lexical, syntactic, semantic, and domain specific features [5]. In the second block, we give the model performance based on BiLSTM, the model uses TENER for character level embedding and the Glove 100d pre-trained embedding for word level embedding [30]. In the third block of, we give the model performance based on Transformer, the model uses TENER for character level embedding and the ELMo [31] for word level embedding. In the last block, we give the experimental result of our proposed model, where data augmentation is “replace all without antonyms”. We can observe that our proposed model outperforms other models. it improves the F1-score from 79.85 to 80.26% on overall performance. Compared with the TENER, our model improves the F1-score from 76.26 to 80.26%. Zhu et al. [7] proposed model on the i2b2 2010 challenge dataset and the result is higher than this article, this is due to the dataset of this article has added three new event types : evidential, occurrence and clinical department, in particular, the evidential and occurrence event types seem more difficult to detect than other event types [1].

The overall results of the model using span F1-score and type accuracy evaluation metrics are shown in Table 4. The first three block are the results of the top three participating in the 2012 i2b2 challenge. The forth block used a combination of supervised, unsupervised and rule-based method, and the task ranked third. First, it identifies event boundaries with a CRF classifier. Then it detects type using separate SVM classifiers [4]. The fifth block, we give the model performance based on Transformer, the Glove 100d pre-trained embedding as word-level embedding and model uses TENER as character-level embedding and encoder. In the last block, we give the experimental result of our proposed model. We can observe that our proposed model Span F1-score is worse than the highest result 1.33%, but our method improves the Type accuracy score from 86 to 93%.

#### Analysis of data augmentation

Data augmentation is a widely used method for dealing with insufficient data. In this section, data augmentation with different degrees methods are used to realize the task of CED. It is based on the TENER model (word-level encoding is Glove 100d pre-trained embedding [29]) and based on our model for comparison experiments. As shown in the Table 5, it is F1-scores in data augmentation methods of a variety of different degrees. Where, “Number” means the number of sentences in the training set after data augmentation, “20%, 40%, 60%, 80%, 100%” means selecting some sentences at random to augment training set, “the Glove 100d” means based on the TENER model, “BioBERT” means based on our model. Comparing the results before and after data augmentation proves that the data augmentation method in this article is effective. Comparing the results of containing antonyms and not containing antonyms, we find that data augmentation without antonyms is more beneficial to the model. Comparing the results produced by replacing one word and replacing all words, we found that only replacing one replaceable word and increasing the training set to 7642 and 10344 sentences can exceed the result of replacing all replaceable words and increasing the training set to 4380 sentences. Therefore, we come to a conclusion,namely,It is more effective to replace all replaceable words without losing performance than replacing only one replaceable word. we find that the prediction result does not increase as the number of sentences in the training set increases. It will reach saturation by a certain order of magnitude. Based on the comprehensive consideration of training speed and performance, the data augmentation degree used in this paper model is “Replace all replaceable words without antonyms”.

**Table 5:** Comparison results of data augmentation, where the bold part indicates the best result

Augmentation method	Number	F1-score (the Glove 100d)	F1-score (BioBERT)
No data augmentation	2250	74.24	76.26
Replace all replaceable words	4393	74.97	80.08
Replace all replaceable words without antonyms	4380	75.28	**80.26**
Replace one replaceable words without antonyms_20%	4935	74.86	79.96
Replace one replaceable words without antonyms_40%	7642	**75.39**	79.96
Replace one replaceable words without antonyms_60%	10344	75.10	80.16
Replace one replaceable words without antonyms_80%	13058	75.12	79.91
Replace one replaceable words without antonyms_100%	15763	75.71	**80.26**

#### Ablation study

We examine the contributions of four main components, namely, data augmentation , BioBERT word-level embedding and TENER character-level embedding and encoder. Tables 6 and 7 show the results, Where “-” means remove component, “$\rightarrow$” means replace component, “TENER$\rightarrow$CNN” means character-level embedding component. “TENER$\rightarrow$BiLSTM” means encoding component. We can observe that data augmentation, BioBERT word-level embedding, TENER character-level embedding and encoder improved the performance of the model to varying degrees. Especially, BioBERT, which has been pre-trained on a large amount of biomedical data and has lots of biomedical information. Data augmentation has a certain impact on model performance, it can alleviate the problem of poor model performance and generalization ability caused by scarcity of data sets. The change of the character-level encoder has less impact on the result, analyze the reason is that the word-level embedding dimension is much smaller than the 768 of the BioBERT word-level embedding, but if the character-level embedding is also adjusted to a larger value, the training speed of the model will be reduced. TENER encoder is also much better than BiLSTM. It increases F1-score by 0.56%. Experimental results show, these four components can help the model learn medical text information better. Data augmentation and BioBERT solve the problem of the small amount of data in the clinical field and the particularity of clinical data.

**Table 6:** Results of ablation study with Precision, Recall and F1-score metrics

Model	Precision	Recall	F1-score
Ours	81.01	0.7953	0.8026
BioBERT $\rightarrow$ Glove 100d	74.84	0.5272	0.6186
-data augmentation	74.54	0.7805	0.7626
TENER $\rightarrow$ CNN	80.96	0.7944	0.8019
TENER $\rightarrow$ BiLSTM	79.95	0.7944	0.7970

“-” means remove component, “$\rightarrow$” means replace component, “TENER $\rightarrow$ CNN” means character-level embedding component. “TENER $\rightarrow$ BiLSTM” means encoding component

**Table 7:** Results of ablation study with Span F1-score and Type accuracy metrics

Model	Span F1-score	Type accuracy
Ours	90.33	93.00
BioBERT $\rightarrow$ Glove 100d	79.08	86.60
-data augmentation	90.01	93.05
TENER $\rightarrow$ CNN	90.28	93.10
TENER $\rightarrow$ BiLSTM	90.13	92.98

“-” means remove component, “$\rightarrow$” means replace component, “TENER $\rightarrow$ CNN” means character-level embedding component. “TENER $\rightarrow$ BiLSTM” means encoding component

#### Error analysis

The prediction performance of different types of events is shown in Table 8, we found that the evidential and occurrence event types seem more difficult to detect than other event types. Especially occurrence event type, there is enough data volume, but the result is much lower than other types. In future work, we will analyze the cause of the low F1-score of the type of event and find ways to solve it to improve the performance of the model.

**Table 8:** Results of different event types

Event type	F1-score	Training	Test
Clinical department	81.64	6.05	5.39
Evidential	75.19	4.49	4.38
Occurrence	66.32	19.95	18.38
Problem	83.75	30.50	31.70
Test	84.09	15.76	15.99
Treatment	84.19	23.25	24.17

## Discussion

There may be two reasons why our model achieves the best performance. On the one hand, the model uses a pre-trained language model (BioBERT) to generate word-level features, which makes the model’s ability to capture word-level features in the medical field relatively strong. On the other hand, a new data enhancement method was proposed and the dataset was expanded, which made the model learn more information. Table 5 shows the results of model testing after using various degrees of data enhancement methods. we come to a conclusion, It is more effective to replace all replaceable words without losing performance than replacing only one replaceable word. And the prediction result will not increase as the number of sentences in the training set increases, it will reach saturation by a certain order of magnitude. The prediction performance of different types of events is shown in Table 8, we found that the occurrence event types seem more difficult to detect than other event types, there is enough data volume, but the result is much lower than other types.

We also found a problem, Examples of data augmentation in Table 1, “worked up” in the sentence is a phrase. If it is split to find and replace, it will change the meaning of the sentence, but it is proved through experiments that it does not affect this paper model for Clinical Event Detection. In order to increase the rationality of the sentence and the performance of the model, in the future We will study how to accurately recognize phrases in sentences for our data augmentation.

## Conclusions

This paper proposes a multi-granularity information fusion encoder-decoder framework. For the first time, this framework applies a TENER model with direction-aware, distance-aware and un-scaled attention to the CED task, it uses the pre-trained language model (BioBERT) to generate word-level features, solves the problem of poor model recognition performance caused by obscure professional terms in electronic medical records. In addition, this paper proposes a new data augmentation method for sequence labeling tasks, which solves the problem of poor model generalization due to insufficient clinical datasets. Experiments on the 2012 i2b2 challenge dataset show that our model achieves superior performance than other existing models. In future work, we will continue to study the reasons for the poor results of the event type and the problem that the meaning of the generated sentence may change due to the occurrence of phrases in the data augmentation.

## Data Availability

The datasets used and analyzed during the current study are available from the first author upon reasonable requests. https://www.i2b2.org/NLP/DataSets/Main.php

## Abbreviations

CED: Clinical Event Detection
BioBERT: A domain-specific language representation model pre-trained on large-scale biomedical corpora
TENER: Adaptive Transformer encoder
NLP: Natural Language Processing
TEs: Temporal expressions
NER: Named entity recognition
SVM: Support Vector Machine
CRF: Conditional Random Field
EDA: Easy data augmentation
GRN: Gated Relation Network
GNNs: Graph Neural Networks
BiLSTMs: Bidirectional long short-term memory network
